# Synthesis of Europium-Doped Fluorapatite Nanorods and Their Biomedical Applications in Drug Delivery

**DOI:** 10.3390/molecules22050753

**Published:** 2017-05-06

**Authors:** Haifeng Zeng, Xiyu Li, Muyang Sun, Sufan Wu, Haifeng Chen

**Affiliations:** 1 Department of Plastic Surgery, Zhejiang Provincial People’s Hospital, People’s Hospital of Hangzhou Medical College, Hangzhou 310014, China; y-zhf@163.com; 2 State Key Laboratory of Oral Diseases, West China Hospital of Stomatology, Sichuan University, Chengdu 610041, China; lixiyu@scu.edu.cn; 3 Department of Biomedical Engineering, College of Engineering, Peking University, Beijing 100871, China; haifeng.chen@pku.edu.cn

**Keywords:** lanthanides, fluorapatite, drug loading, nano carrier

## Abstract

Europium (Eu)-doped fluorapatite (FA) nanorods have a biocompatibility similar to that of hydroxyapatite (HA) for use as cell imaging biomaterials due to their luminescent property. Here, we discuss the new application of europium-doped fluorapatite (Eu-FA) nanorods as an anticancer drug carrier. The Eu-FA nanorods were prepared by using a hydrothermal method. The morphology, crystal structure, fluorescence, and composition were investigated. The specific crystal structure enables the effective loading of drug molecules. Doxorubicin (DOX), which was used as a model anticancer drug, effectively loaded onto the surface of the nanorods. The DOX release was pH-dependent and occurred more rapidly at pH 5.5 than at pH 7.4. The intracellular penetration of the DOX-loaded Eu-FA nanorods (Eu-FA/DOX) can be imaged in situ due to the self-fluorescence property. Treatment of melanoma A375 cells with Eu-FA/DOX elicited a more effective apoptosis rate than direct DOX treatment. Overall, Eu-FA exhibits potential for tracking and treating tumors and may be potentially useful as a multifunctional carrier system to effectively load and sustainably deliver drugs.

## 1. Introduction

Fluorapatite (FA) is one of the inorganic constituents of bone or teeth and is used for hard tissue repairs and replacements [1]. FA(Ca_10_(PO_4_)_6_F_2_) can be synthetically prepared from hydroxyapatite and contains a suitable molecular concentration of OH- groups and F^−^ ions [2]. Nanophase hydroxyapatite or fluorapatite do not have fluorescence characteristics; in order to obtain these properties, the luminescent center should be added. Some scholars tend to integrate HA with fluorescein isothiocyanate (FITC) to label and track HeLa cells [3]. However, FITC demonstrates a short quenching time and poor stability in vivo. When doped with lanthanides (Eu^3+^ Tb^3+^ or Gd^3+^), this material exhibits special photoluminescence and has the potential for application in biomedical studies [4,5,6]. Some lanthanide ion-doped materials (Eu^3+^ or Tb^3+^) can emit visible fluorescence (i.e., green or red) when excited by ultraviolet or blue excitation wavelengths. In comparison to fluorescent dyes or semiconductor quantum dots (QDs), these lanthanide-doped nanoparticles are advantageous due to their photostability, sharp visible emission bandwidth, and nontoxicity [7,8,9]. In previous cell labeling and tracking studies, lanthanide ion-doped fluorapatite exhibited long excited state lifetimes and no toxicity to bone marrow mesenchymal stem cells [10,11].

Their high surface-to-volume ratio, reactivities, and biocompatibility make FA nanorods favorable for use in drug delivery studies [12,13]. In this study, europium-doped fluorapatite (Eu-FA) nanorods were prepared and characterized. In addition, the obtained multifunctional Eu-FA nanorods were employed as a drug delivery carrier to investigate their drug loading and release properties using doxorubicin (DOX) as a model drug. Melanoma A375 cells were treated with DOX-loaded Eu-FA nanorods (Eu-FA/DOX) to confirm that Eu-FA/DOX induced apoptotic behavior in the tumor cells. The loading capacity of DOX and its release profile were investigated, and the in vitro biological efficacy of the intracellular delivery system was confirmed.

## 2. Results and Discussion

**Figure 1:**
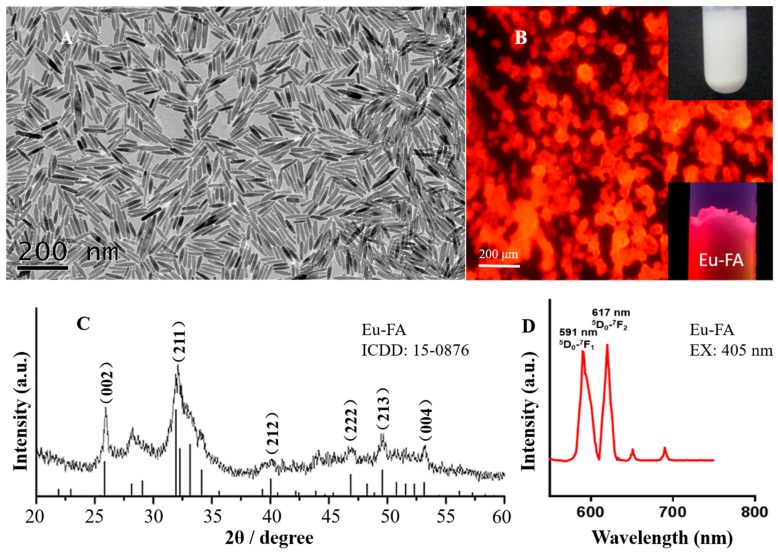
(**A**) TEM results for the europium-doped fluorapatite (Eu-FA) crystals indicate a rod-like morphology; (**B**) Eu-FA nanorod powder under UV light and fluorescence microscopy; (**C**) XRD patterns correspond to a classical hexagonal phase; (**D**) Emission intensity of Eu-FA excited at 405 nm.

**Scheme 1:**
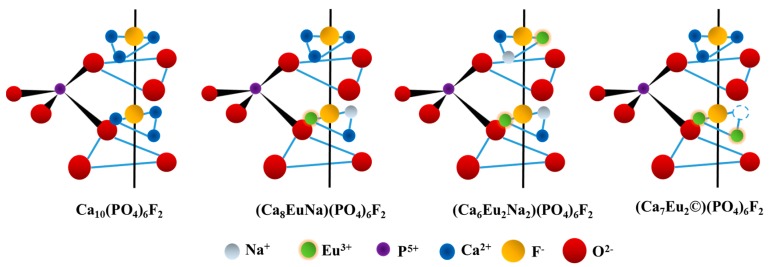
Schematic diagram of different Eu-doping lattice models (Ca_10_(PO_4_)_6_F_2_, (Ca_8_EuNa)(PO_4_)_6_F_2_, (Ca_6_Eu_2_Na_2_)(PO_4_)_6_F_2_, (Ca_7_Eu_2_©)(PO_4_)_6_F_2_, © means Ca^2+^ vacancy) along the fluorine channel of the FA crystal structure.

The TEM results indicate that the Eu-FA crystals are on the nanoscale with a rod-like morphology (Figure 1A). The TEM image also indicates that the Eu-FA nanorods are straight with a uniform morphology and good crystallinity. The diameters of the Eu-FA nanorods are 20–40 nm, with lengths ranging from 100 to 170 nm. Figure 1B shows the nanoparticle powders under UV light in an Eppendorf tube and a converted fluorescence microscope. Figure 1C shows the X-ray diffraction patterns (XRD) of the synthesized Eu-FA nanorods. All of the diffraction peaks can be indexed to the classical hexagonal phase according to ICDD 15-0876. The sharp characteristic peaks at approximately 25.91 and 31.91 correspond to the (002) and (211) lattice planes. Figure 1D indicates that the emission intensity of Eu-FA is 591 (^5^D_0_-^7^F_1_) nm and 617 nm (^5^D_0_-^7^F_2_) upon excitation at 405 nm. Doping of Eu^3+^ ions substitute part of the Ca^2+^ ions in the Eu-FA crystals. The proposed lattice models (Scheme 1) show several mechanisms by which Eu^3+^ ions incorporate into the apatite lattice, which is important for understanding the mechanism of synthesis of Eu-FA nanorods. The F^−^ ions in the FA crystal occupy a smaller space and have a weaker steric hindrance compared to the –OH groups in the HA crystal. In other words, the F^−^ ions have a stronger interaction with Eu^3+^ ions than –OH groups [11,14].

**Figure 2:**
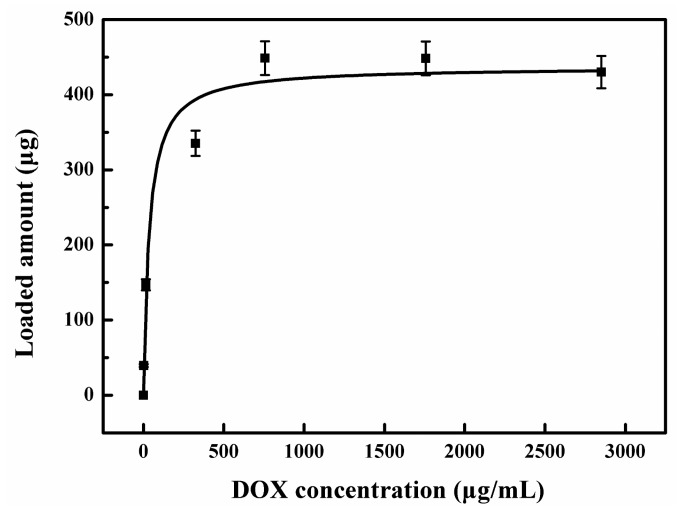
Doxorubicin (DOX) loading curve for Eu-FA, which was obtained by measuring the amount of DOX loaded onto the nanorods.

The DOX loading was recorded at a given nanocarrier content while varying the DOX concentrations from 0 to 5000 μg/mL. The DOX loading amount increased gradually as the DOX concentration increased, and a maximum loading of 43.7% was achieved (Figure 2). The DOX molecules can be adsorbed onto the surface of the Eu-FA nanorods. DOX is a small molecule and can thus possibly be incorporated within the mesoporous or the hollow space. The high surface-to-volume ratio and the unique crystal structure make the Eu-FA nanorods easy to absorb DOX molecules. In addition, DOX has a pKa of 8.3 and is positively charged at a pH of 7.4. Therefore, DOX is attracted to the negatively charged nanocarriers without additional surface functionalization [15]. As DOX is oppositely charged to the Eu-FA surface and is small in size, its incorporation into the mesoporous should be easy, and even penetration into the hollow space of the nanorods should be possible [16].

**Figure 3:**
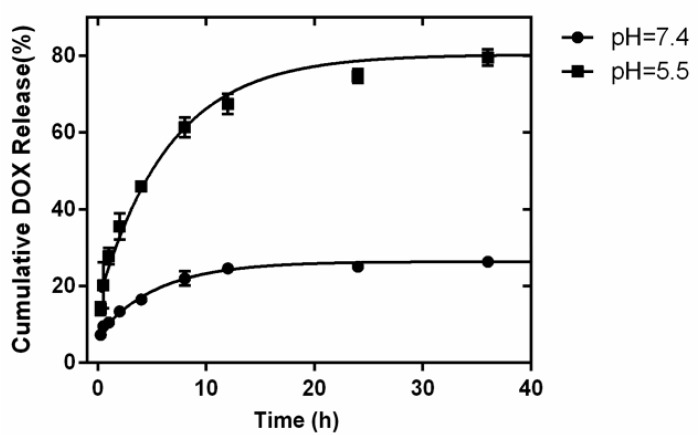
DOX release with different pH values (pH = 5.5, 7.4).

The loaded DOX was subsequently released into PBS with different pH values. Although a pH of 7.4 is representative of normal physiological conditions, an acidic pH of 5.5 is shared by the extracellular tissues of tumor cells [17]. The DOX molecules exhibited a significant pH-dependent release (Figure 3). This pH-sensitive phenomenon may be due to an increase in the hydrophilicity and solubility of DOX in acidic environments due to the stronger protonation of the –NH_2_ groups in the DOX molecules [18]. The DOX molecule contains various active and functional groups (such as –C=O, –COCH_2_OH, –OH, and –NH_2_), so it can easily inter-convert from one functional group to other by changing the pH value of the solution [19,20]. It is also easier to degrade the fluorapatite in an acid environment to promote DOX release. These reasons may contribute to why DOX has a faster release rate at pH 5.5 than at pH 7.4. Low drug release efficacy is favorable for drug delivery, and the high DOX release rate in acidic environments is beneficial for killing tumor cells. The results of the drug release tumor cell apoptosis test confirmed this viewpoint.

**Figure 4:**
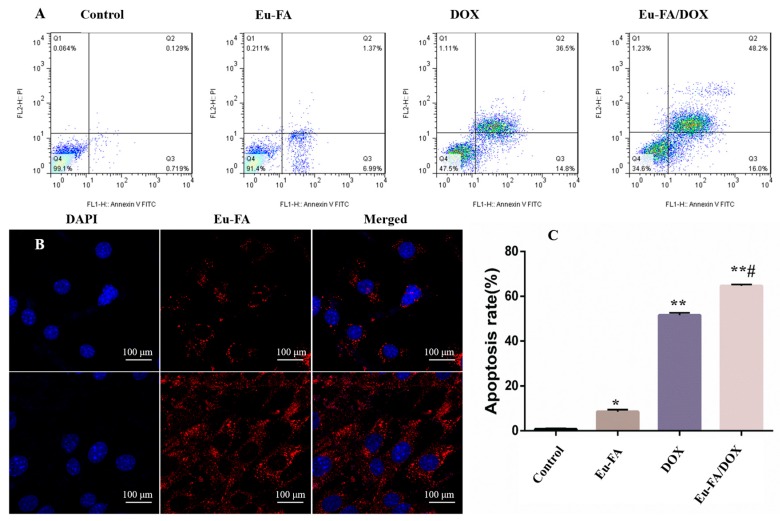
(**A**) Flow cytometry results for apoptosis after the A375 cells were treated with the control, Eu-FA (40 μg/mL), DOX (17.48 μg/mL) and Eu-FA/DOX (40 μg/mL Eu-FA full loading with DOX); (**B**) Blue fluorescence from DAPI and red fluorescence from Eu-FA were observed in the cytoplasm or within the nucleus; (**C**) Statistical results of apoptosis in each group (*# means *p* < 0.05, ** means *p* < 0.01).

Next, the cellular uptake of Eu-FA was examined. The A375 cells were treated with 10 μg of Eu-FA nanorods for 4 h. The intracellular penetration of Eu-FA/DOX can be imaged in situ due to the self-fluorescence property. The location of the Eu-FA nanorods in the A375 cells was confirmed by confocal laser scanning microscopy. The red regions represent the fluorescence signal of Eu-FA, and the blue signals arise from the DAPI-stained nucleus. The red fluorescence signals of Eu-FA were observed in the cytoplasm (around the blue signals) or within the nucleus (coincident with the blue signals) of the A375 cells treated with DOX (Figure 4B). Therefore, only 4 h of treatment resulted in rapid uptake of the Eu-FA nanorods by the cells.

To investigate the apoptosis rate induced by Eu-FA/DOX, the treated cells were double-stained with FITC-Annexin V and PI followed by analysis with flow cytometry. Propidium iodide (PI) was used in conjunction with Annexin V to determine if the cells were viable, apoptotic, or necrotic from differences in the plasma membrane integrity and permeability. This approach is a universal and accurate method for detecting cell apoptosis [21]. After a period of incubation, tumor cells will produce an acidic environment, and the pH value of the solution will undergo a dynamic change process and eventually attain a weak acidity. This acidic environment is conducive to the release of DOX in Eu-FA/DOX [22]. As shown in (Figure 4A,C), few apoptotic cells were detected in the untreated control. However, cell apoptosis was induced in the groups treated with DOX and Eu-FA/DOX (i.e., approximately 50.9% and 64.1%, respectively). Interestingly, more apoptosis cells were observed with the Eu-FA/DOX treatment group compared to that with the DOX-only treatment group, and this difference was statistically significant. This means more apoptosis was observed when DOX was uploaded by the Eu-FA nanocarrier system. Therefore, in the group treated with Eu-FA, 8.31% of the population exhibited apoptotic cells. The non-apoptotic cell fraction was considered to be necrotic (i.e., dying from a cause other than apoptosis). At this point, the cell death mechanism of the A375 cells treated with DOX is considered to be different from that of cells treated with Eu-FA/DOX. 

## 3. Materials and Methods

### 3.1. Synthesis of Eu-FHA Nanorods

Eu^3+^-doped FHA nanoparticles were synthesized via a hydrothermal method (synthesized by the Institute of Biomedical Engineering, Peking University). All chemicals were analytical grade. Exactly 0.5 g of octadecylamine were dissolved in 4 mL of oleic acid while heating, and 16 mL of ethanol and an aqueous solution of Ca(NO_3_)_2_ (0.28 M, 7.0 mL) were then added while stirring. NaF (0.24 M, 2.0 mL), Eu(NO_3_)_3_ (0.20 M, 2.0 mL), and Na_3_PO_4_ (0.20 M, 7.0 mL) were added to the solution followed by an additional 5 min of stirring. Then, the sealed flask was heated at 160 °C for 16 h. The obtained mixture consisted of FA nanoparticles doped with Eu^3+^, and these nanoparticles were collected by centrifugation and freeze-dried.

### 3.2. Characterization of Eu-FHA Nanorods

Transmission electron microscopy (TEM) was carried out on a JEM-2100 instrument to determine the sizes and morphologies of the Eu-FHA nanorods. The samples were prepared by placing a drop of a dilute ethanol dispersion of the products on the surface of a copper grid. XRD analysis was performed using an Image Plate X-ray Diffractometer (RAPID-S, Rigaku, Japan) with Cu Ka radiation. The luminescence spectra were recorded on a Hitachi F-4500 fluorescence spectrophotometer. Luminescent photography of the sample was performed under UV light at 390 nm. 

### 3.3. Preparation of the Drug Loading and Release System

The DOX loading of Eu-FA nanorods were prepared using an absorption method. Doxorubicin hydrochloride (DOX, Shanghai Sangon, Shanghai, China) was dissolved in the PBS solution at pH 7.4 to produce stock solutions with concentrations of 0, 200, 750, 2000, 3000, 4000, and 5000 μg/mL. Five milligrams of the Eu-FA sample were ultrasonically dispersed in each DOX solution for 5 min, and then kept in a 37 °C water bath for 12 h. To quantify the drug-loading amount, the nanorods were centrifuged at 10,000 rpm for 5 min, and the supernatant was gathered and detected by UV-vis spectrophotometer at an absorbance of 483 nm.

To study the release curve of DOX from the nanocarrier samples, the as-prepared Eu-FA/DOX nanorods were used. A 2 mg sample was dispersed in a 2 mL PBS solution (0.01 M, pH 7.4 and 5.5), followed by dialysis (3500 Dalton) in an air bath oscillator at 37 °C. The dialysis bag was placed in a buffer medium of 8 mL, and the vibration was slow under the condition of 37 °C. At the different time points (15 min, 30 min, 2 h, 4 h, 8 h, 12 h, 1 h, 24 h, 36 h), the amount of drug released into the medium was measured by using a UV-Vis spectrophotometer with an absorbance of 483 nm, and the release rate was then calculated. 

### 3.4. Cell Culture and Fluorescence Imaging

Melanoma A375 cells were seeded at 10^5^ cells in each well of 6-well culture plates and treated with Eu-FA nanorods at a concentration of 10 μg/mL for 4 h. The fluorescent images were observed and analyzed using a laser scanning confocal microscope (LSM780, Carl Zeiss, Dresden, Germany). The cells were counterstained with DAPI (Invitrogen, Carlsbad, CA, USA) to observe the nucleus.

### 3.5. Cell Apoptosis Detection

The A375 cells treated with Eu-FA, DOX, or Eu-FA/DOX for 24 h were harvested. The concentration of the Eu-FA was 40 μg/mL, the DOX concentration was 17.48 μg/mL, which was calculated by the maximum drug loaded amount at the adsorption value of 43.7%, and the initial pH value of the solution was 7.4. Flow cytometry was employed to detect cell death and apoptosis according to the manufacturer’s instruction. After being harvested via trypsinization and lysed with ice-cold PBS, the cocultured cells with Annexin V-FITC (50 μg/mL, Sigma, Saint Louis, MO, USA) were incubated for 15 min in a dark at room temperature. Then, the re-suspended cells were stained with propidium iodide (10 μg/mL, Sigma). A Becton Dickinson FACS/Calibur cytometer (BD Biosciences, Franklin Lakes, NJ, USA) was employed to analyze the Annexin V-FITC and propidium iodide signals.

### 3.6. Statistics Analysis

Statistical analysis was performed using the SPSS 22.0 software. Student’s *t*-tests were performed to determine the statistical significance between experimental groups. *p* < 0.05 was considered to indicate statistical significance. All results are expressed as the mean ± standard.

## 4. Conclusions

In summary, the as-synthesized Eu-FA nanorods exhibited excellent photoluminescence and high drug-loading capacity. We believe that this multifunctional nanocarrier will generate further interest and open new avenues for research on the applicability of using these nanoparticles in cancer treatment.
